# Organ-specific *PTB1-*associated microRNAs determine expression of pyruvate kinase isoforms

**DOI:** 10.1038/srep08647

**Published:** 2015-02-27

**Authors:** Kohei Taniguchi, Yuko Ito, Nobuhiko Sugito, Minami Kumazaki, Haruka Shinohara, Nami Yamada, Yoshihito Nakagawa, Tarou Sugiyama, Manabu Futamura, Yoshinori Otsuki, Kazuhiro Yoshida, Kazuhisa Uchiyama, Yukihiro Akao

**Affiliations:** 1 United Graduate School of Drug Discovery and Medical Information Sciences, Gifu University, 1-1 Yanagido, Gifu 501-1193, Japan; 2 Department of General and Gastroenterological Surgery, Osaka Medical College, Daigaku-machi, Takatsuki, Osaka 569-8686, Japan; 3 Department of Anatomy and Cell Biology, Division of Life Sciences, Osaka Medical College, 2-7 Daigaku-machi, Takatsuki, Osaka 569-8686, Japan; 4 Department of Gastroenterology, Fujita Health University, School of Medicine, Kutsukake-cho, Toyoake, Aichi 470-1192, Japan; 5 Department of Oncological surgery, Gifu University School of medicine, 1-1 Yanagido, Gifu 501-1193, Japan

## Abstract

The Warburg effect is a well-known feature of cancer cells. However, its' functional significance hasn't been elucidated yet. Pyruvate kinase muscle (PKM), which is a rate-limiting glycolytic enzyme, has 2 isoforms, PKM1 and PKM2. It has been reported that PKM2 is a tumor-specific isoform and promotes the Warburg effect. Also, it has been thought that tumor cells switch their PKM isoform from PKM1 to PKM2 during tumor development. Here, we showed that this switching machinery was induced only in limited cases, based on PKM expression in normal tissues, and that brain-specific microRNA (miR)-124 and muscle-specific miR-133b regulated this machinery by controlling PKM expression through targeting polypyrimidine tract-binding protein 1 (*PTB1*), which is a splicer of the *PKM* gene. Also, we confirmed that the PKM2/PKM1 ratio was further elevated in other PKM2-dominant organs such as colon through the down-regulation of these *PTB1*-associated microRNAs during tumor development.

Cancer cell growth entails numerous metabolic changes. Cancer cells exhibit a metabolic phenotype characterized by increased glycolysis, regardless of oxygen availability—a phenomenon termed the Warburg effect^1^,^2^. The reason why cancer cells undergo the Warburg effect is not entirely understood. However, it is well accepted that the metabolic change due to the Warburg effect increases the levels of metabolic intermediates that are required for the synthesis of biological macromolecules^3^ and avoids the cell death induced by ROS^4^. This change is partly achieved through control of the expression of pyruvate kinase muscle (PKM) isoforms, which are rate-limiting glycolytic enzymes. PKM has 2 isoforms, PKM1 and PKM2, which are produced by alternative splicing of transcripts in the *PKM* gene. *PKM1* contains exon 9 and lacks exon 10, whereas *PKM2* contains exon 10 and lacks exon 9^5^. The alternative splicing that generates the *PKM2* mRNA transcript is reported to be mediated by members of the heterogeneous nuclear ribonucleoprotein (hnRNP) family, such as polypyrimidine tract-binding protein 1 (PTB1, also known as hnRNPI)^6^,^7^.

MicroRNAs (miRNA; miR) have emerged recently as a large group of short (18–25 nucleotides), non-coding, small RNA molecules that negatively regulate gene expression^8^,^9^. Although the specific biological functions of most miRNAs remain largely unknown, there is increasing experimental evidence supporting the role of miRNAs in the regulation of a wide range of physiological or pathophysiological responses, including development^10^, cellular apoptosis^11^, differentiation^12^, cell proliferation^13^, and cancer^14^,^15^,^16^. In addition, a recent study suggests that some miRNAs target hnRNP family members including *PTB1* and thereby regulate the Warburg effect^17^.

PKM2 is exclusively expressed in embryonic, proliferating, and cancer cells, and promotes the Warburg effect^18^. Recent studies suggest that heightened expression of PKM2 is critical for the maintenance of cancer cell growth and is associated with a poor prognosis in some types of cancer^19^,^20^. On the other hand, it was previously thought that PKM1 is expressed in normal differentiated tissues and that the switching of PKM expression from PKM1 to PKM2 occurs during cancer development^18^,^21^. However, a few recent studies demonstrated no evidence for the change of PKM1 to PKM2 during the development of certain cancers^22^,^23^. The mechanisms underlying this change in expression profile have not been elucidated yet. Here, we demonstrated that the switching of PKM isoform expression from PKM1 to PKM2 during cancer development occurred only in limited types of tumors, as based on PKM expression profiles in their normal tissues. In addition, we found that PKM expression profiles in human organs and the switching of PKM isoform expression during cancer development were regulated by *PTB1*-associated microRNAs such as miR-124 specific to neuronal cells and miR-133b specific to muscle cells, thus further establishing the Warburg effect.

## Results

### PKM2 is dominant in all proliferating cells and PKM1 is dominant only in normal tissues from high energy-demanding organs

**Figure 1:**
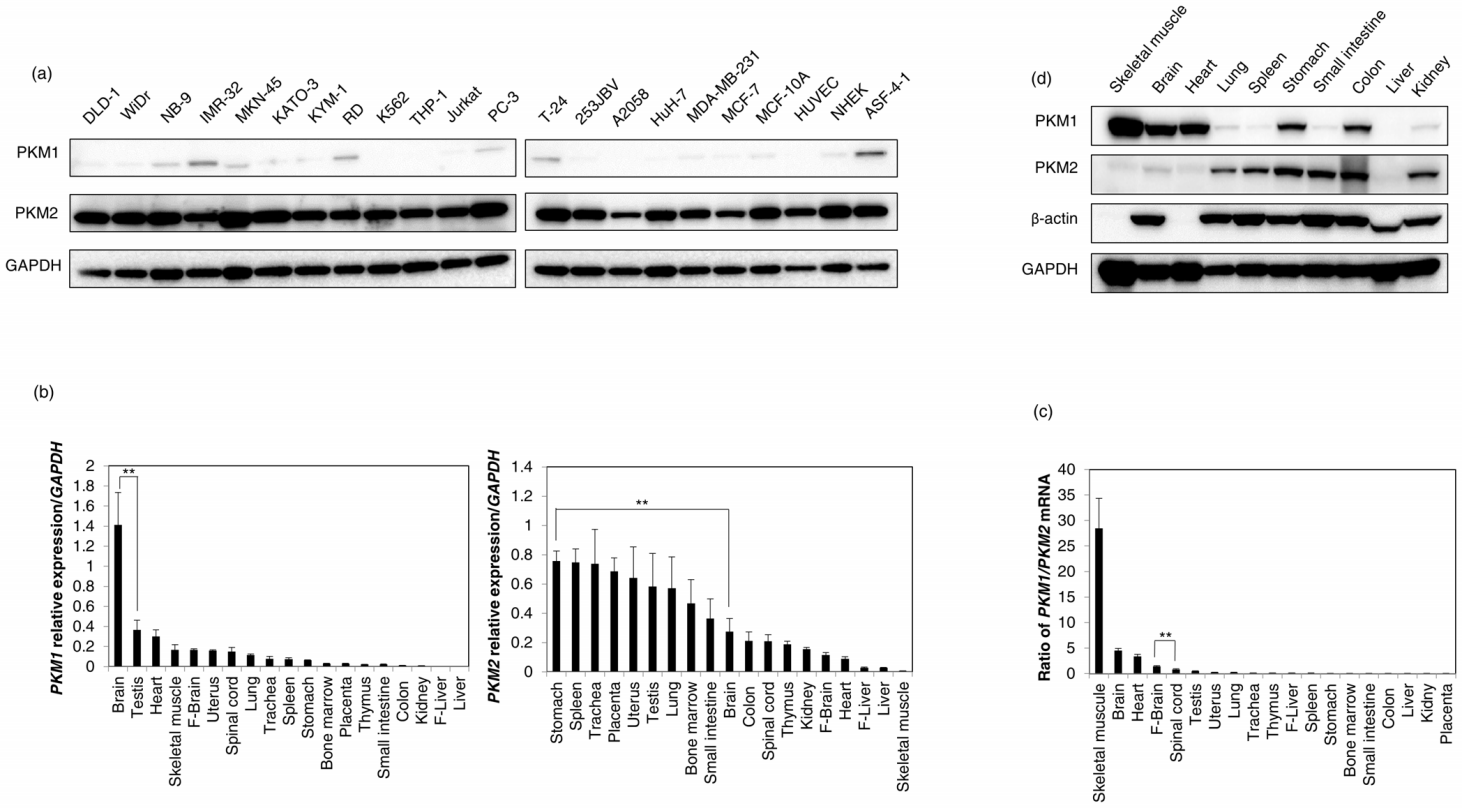
(a) Expression profile of PKM isoforms in various cancer cell lines, control cell lines, and cells in primary culture. DLD-1 and WiDr, human colon cancer cell lines; NB-9 and IMR-32, human neuroblastoma cell lines; MKN-45 and KATO-3, human gastric cancer cell lines; KYM-1 and RD, human rhabdomyosarcoma cell lines; K562, human chronic myelogenous leukemia cell line; THP-1, human acute monocytic leukemia cell line; Jurkat, human T-cell leukemia cell line; PC-3, prostate cancer cell line; T-24 and 253JBV, human bladder cancer cell lines; A2058, human malignant melanoma cell line; HuH-7, human liver cancer cell line; MDA-MB-231 and MCF-7, human breast cancer cell lines; MCF-10A, human breast epithelial cell line; HUVEC, human umbilical vein endothelial cells; NHEK, human epidermal keratinocyte cells; ASF-4-1, human normal diploid fibroblast cells. PKM1 and PKM2 were detected by Western blotting under the same experimental conditions at the same time. The full-length blots are presented in Supplementary Figure S2a. (b) Relative expression of *PKM1* and *PKM2* mRNAs in various human normal tissues. F-brain, Fetal brain; F-liver, Fetal liver. (c) *PKM1/PKM2* ratio of those cases. (d) Expression profile of PKM isoforms in tissues from mouse organs. PKM1 and PKM2 were detected by Western blotting in under the same experimental conditions at the same time. The full-length blots are presented in Supplementary Figure S2b. Results are presented as the mean ± SD (** *P* < 0.01).

A growing body of evidence indicates that PKM2 is important for cancer metabolism and tumor growth^18^,^24^,^25^. Therefore, we firstly examined the protein expression profile of PKM isoforms in various cell lines. As a result, PKM2 was shown to be dominant in all human cancer cell lines tested (Fig. 1a). Unexpectedly, PKM2 was also the dominant one even in control cell lines such as a breast epithelial cell line (MCF-10A) and human primary cells such as human umbilical vein endothelial cells (HUVEC), normal human epidermal keratinocyte (NHEK) and normal diploid fibroblast cells (ASF-4-1; Fig. 1a). Next, we examined the mRNA expression profiles of PKM isoforms in human normal tissues. Surprisingly, PKM1 was dominant in only skeletal muscle, brain, heart, and fetal brain; whereas in all of the other normal tissues tested, PKM2 was dominant (Fig. 1b, c). Thus, to validate the protein expression profiles of PKM isoforms in normal tissues, we examined the expression profiles of such isoforms in the tissues from mouse organs. As a result, PKM1 was dominant in only brain, skeletal muscle, and heart (Fig. 1d), as was the case for human normal tissues at the mRNA level. Also, there was no difference in the expression levels of PKM1/2, as detected with the anti-PKM1/2 in some representative samples (Supplementary Fig S1a). These findings suggest that in normal tissues, PKM1 was the dominant isoform expressed only in high energy-demanding organs such as brain and muscle.

### PTB1-associated microRNAs contributes to the regulation of the PKM isoform expression in an organ-dependent manner

**Figure 2:**
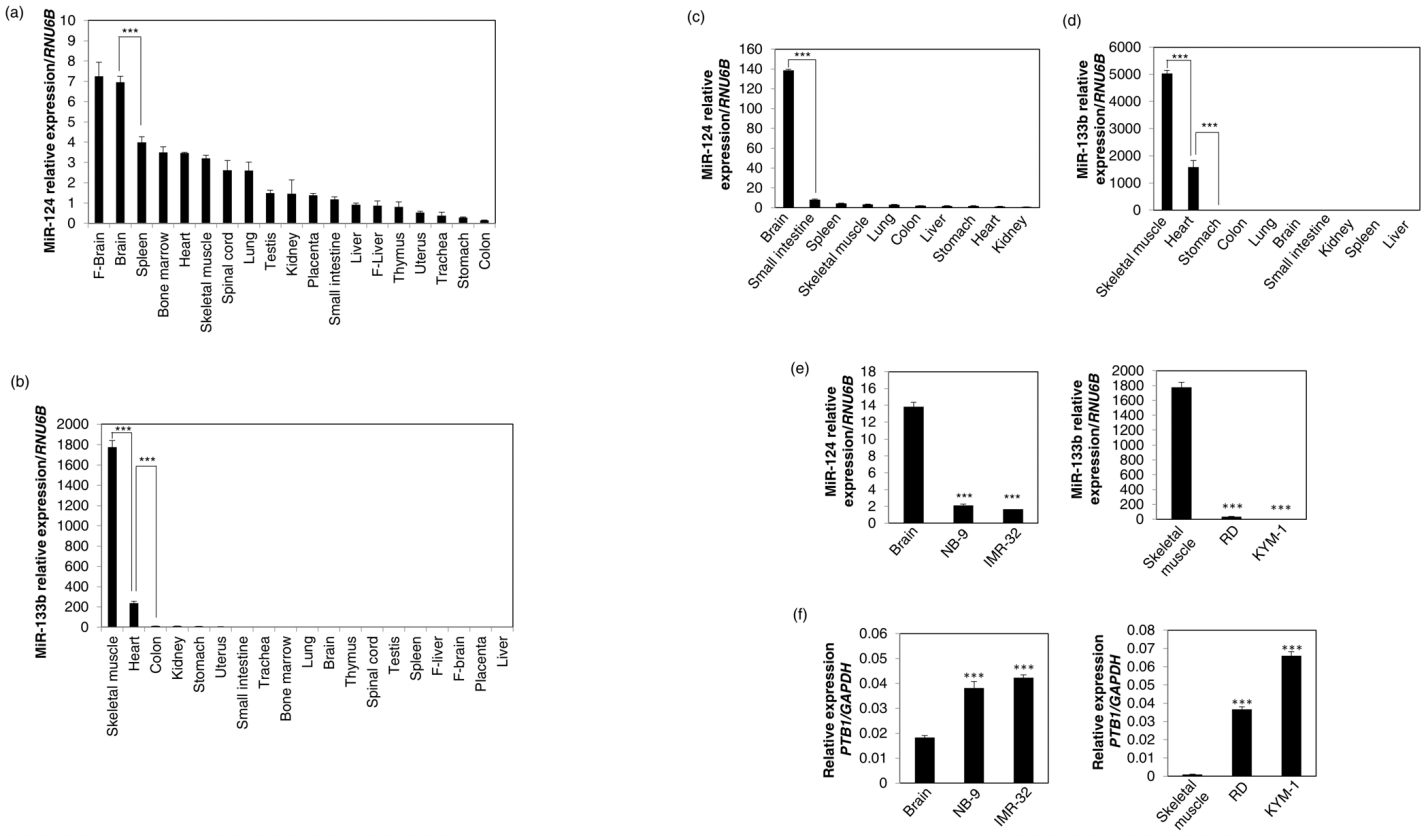
(a) Relative expression levels of miR-124 in various human normal tissues. F-brain, Fetal brain; F-liver, Fetal liver. (b) Relative expression levels of miR-133b in various human normal tissues. (c) Relative expression levels of miR-124 in mouse organs. (d) Relative expression levels of miR-133b in mouse organs. (e) Relative expression levels of miR-124 in normal brain tissue and neuroblastoma cell lines NB-9 and IMR-32 and those of miR-133b in normal skeletal muscle tissue and rhabdomyosarcoma cell lines RD and KYM-1. (f) Relative expression levels of PTB1 in normal brain or skeletal muscle tissue and neuroblastoma and rhabdmyosarcoma cell lines. Results are presented as the mean ± SD (*** *P* < 0.001).

PKM expression is regulated by PTB1, which is one of the heterogeneous nuclear ribonucleoprotein (hnRNP) proteins^6^,^21^. *PTB1* is targeted by several miRNAs such as miR-1, miR-9, miR-124, miR-133, and miR-137, based on data in the Target Scan 6.2 database (http://www.targetscan.org/) in miRBase (http://www.mirbase.org/). Among the *PTB1*-associated miRNAs, miR-124, miR-9, and miR-137 are known as brain-specific miRNAs^26^,^27^,^28^,^29^,^30^; whereas miR-1 and miR-133 are muscle-specific ones^31^,^32^,^33^. Among them, we examined the expression profiles of miR-124 and miR-133b, because both miR-124 and miR-133b have 2 target sites each in the 3′UTR of *PTB1*. As a result, the expression levels of miR-124 were preferentially higher in the tissues from brain, skeletal muscle, and heart than in those from the other organs tested (Fig. 2a). On the other hand, the expression levels of miR-133b were preferentially higher in the tissues from skeletal muscle and heart compared with those from the other organs tested (Fig. 2b). These tissues are found in PKM1-dominant, high energy-demanding organs, as stated above. In addition, we examined the expression profiles of miR-124 and miR-133b in tissues from mouse organs. As a result, the expression level of miR-124 was preferentially higher in brain (Fig. 2c); whereas the expression levels of miR-133b were preferentially higher in skeletal muscle and heart (Fig. 2d), as were the case for human normal tissues. These findings taken together imply that these *PTB1*-associated miRNAs contributed to the regulation of the PKM isoforms expression in an organ-dependent manner.

### PTB1-associated miRNAs relate to cancer development in specific tissues

Next, we examined the involvement of *PTB1*-associated miRNAs in the maintenance of cancer proliferation. Much evidence indicates that PKM2 expression is up-regulated in typical brain tumors such as glioblastoma and glioma cells^7^,^34^,^35^,^36^. Therefore, we used NB-9 and IMR-32 neuroblastoma cells to confirm the consistency of this tendency in brain tumor cells. As shown in Fig. 2e, brain-specific miR-124 was down-regulated in both neuroblastoma cell lines as compared with its level in normal brain. Furthermore, muscle-specific miR-133b was extremely down-regulated in rhabdomyosarcoma cell lines RD and KYM-1, compared with its level in normal skeletal muscle (Fig. 2e). In addition, the expression of PTB1 was up-regulated corresponding to the down-regulation of these miRNAs (Fig. 2f), which likely would lead to the maintenance of the Warburg effect^6^,^7^. These findings suggest that *PTB1*-associated miRs-124 and -133b were related to cancer development and proliferation, especially in PKM1-dominant organs such as brain and muscle.

### PTB1-associated miRNAs trigger the shift of PKM isoform expression from PKM2 to PKM1 through the down-regulation of PTB1

**Figure 3:**
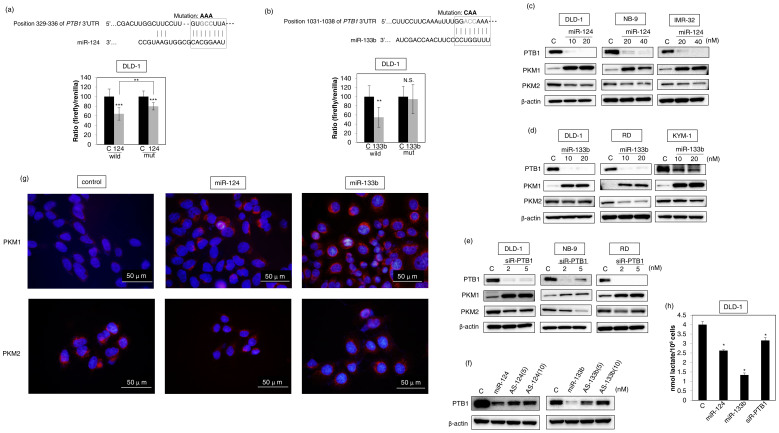
(a) Luciferase activities after co-transfection of DLD-1 cells with control or miR-124 (wild-type or mutant-type) pMIR vectors having the predictive miR-124 binding site in the 3′UTR of *PTB1*. The upper panel shows the region of the 3′-UTR of human *PTB1* mRNA complementary to the mature miR-124. The box indicates the predicted binding sites for miR-124. (b) Same as “a” except miR-133b was used. (c) Expression of PTB1, PKM1, and PKM2 proteins at 72 h after the transfection of DLD-1, NB9 or IMR-32 cells with miR-124 (10, 20 or 40 nM). (d) Expression of PTB1, PKM1, and PKM2 proteins at 72 h after transfection of DLD-1, RD or KYM-1 cells with miR-133b (10, 20 nM). (e) Expression of PTB1, PKM1, and PKM2 proteins at 72 h after transfection of DLD-1, NB-9 or RD cells with siR-PTB1 (2, 5 nM). (f) Effect of combined treatment of DLD-1 cells with antagomiR-124 and miR-124 or antagomiR-133b and miR-133b. DLD-1 cells were transfected with non-specific control, miR-124/miR-133b (10 nM), miR-124/miR-133b (10 nM) + antagomiR-124/antagomiR-133b (5 nM) or miR-124/miR-133b (10 nM) + antagomiR-124/antagomiR-133b (10 nM). The expression level of PTB1 was assessed at 48 h after the transfection. The full-length blots are presented in Supplementary Figure S3a. (g) Immunofluorescence of PKM1 (upper panels) and PKM2 (lower panels) at 48 h after transfection of DLD-1 cells with miR-124 (20 nM) or miR-133b (20 nM). Left panels, treatment with control miRNA; middle panels, treatment with miR-124; right panels, treatment with miR-133b. PKM1 or PKM2 is stained red, and nuclei are stained blue. (h) Lactate production was measured at 48 h after the transfection of DLD-1 cells with miR-124 (20 nM), miR-133b (20 nM) or siR-PTB1 (5 nM). Results are presented as mean± SD (* *P* < 0.05; ** *P* < 0.01; *** *P* < 0.001; N.S., not statistically significant).

Next, we examined whether these miRNAs regulated the PKM isoform expression through the targeting of *PTB1*. Firstly, to validate whether these miRNAs targeted *PTB1*, we performed a luciferase activity assay for *PTB1*. As a result, the luciferase reporter activity of wild-type pMIR-PTB1 was significantly inhibited after the introduction of miR-124 or miR-133b into DLD-1 cells (Fig. 3a, b). On the other hand, mutation of the *PTB1* 3′-UTR-binding site markedly abolished the ability of either miRNA (Fig. 3a, b). Secondly, by performing Western blot analysis we examined the protein expression of PTB1, PKM1, and PKM2 after the transfection of DLD-1, NB-9, and IMR-32 cells with miR-124 and after that of DLD-1, RD, and KYM-1 cells with miR-133b. As a result, Western blot analysis indicated that when PTB1 was down-regulated, PKM isoform expression was shifted from PKM2 to PKM1 in all cells tested that had been transfected with either miRNA (Fig. 3c, d). Similarly, knockdown of *PTB1* up-regulated PKM1 and down-regulated PKM2 in DLD-1, NB-9 or RD cells (Fig. 3e). Furthermore, the treatment with antagomiR-124 or antagomiR-133b significantly reversed the expression level of PTB1 in DLD-1 cells (Fig. 3f). Moreover, to validate whether these observations would hold at the single-cell level, we performed immunofluorescence (IFC) using DLD-1 cells that had been transfected with either miRNA. As a result, immunostaining for PKM1 showed significantly increased intensity in the treated cells at the single-cell level. On the other hand, PKM2 expression was slightly decreased (Fig. 3g). Therefore, the PKM1/PKM2 ratio was also remarkably elevated at the single-cell level. Finally, we examined the lactate production by DLD-1 cells after transfection of them with miR-124, miR-133b or siR-PTB1. The lactate production was remarkably suppressed in all cases (Fig. 3h). These findings taken together suggest that these *PTB1*-associated miRNAs contributed to shifting of PKM isoform expression from PKM2 to PKM1 through the down-regulation of PTB1 at the translational level and thereby affected the Warburg effect.

### The level of PKM2 expression is further elevated in tumors from PKM2-dominant organs

**Figure 4:**
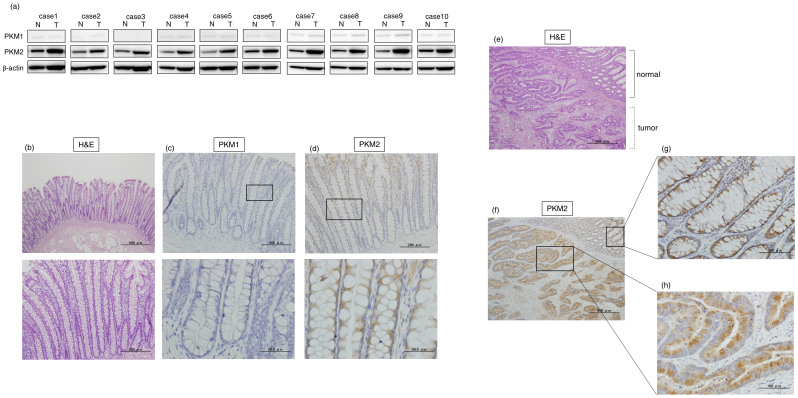
Expression of PKM1 and PKM2 in clinical colorectal cancer samples. (a) The protein expression of PKM1 and PKM2 in clinical specimens of cancer tumor (T) and the adjacent normal tissues (N) is shown. PKM1 and PKM2 were detected by Western blotting in under the same experimental conditions at the same time. The full-length blots are presented in Supplementary Figure S3b. (b–d) Immunohistochemical staining of normal colon tissue adjacent to tumor tissue of case 10. Results of H&E staining (b), staining with anti-PKM1 antibody (c), and staining with anti-PKM2 (d) are shown. The boxed regions in “c” and “d” are enlarged in the images below. (e–h) Immunohistochemical staining of clinical colorectal cancer tissue specimen of representative case 3. H&E-stained section with normal tissue (upper right corner) neighboring the tumor area in the section is shown (e), along with the same section stained with anti-PKM2 antibody (f). Enlarged views of boxed areas in “f” show normal colorectal crypt in mucosa (g) and tumor area (h) stained with anti-PKM2 antibody.

In order to examine the expression profiles of PKM isoforms in the tumor samples from PKM2-dominant organ tissues, we investigated the expression levels of miRs-124, -133b, and PKM isoforms in 10 clinical colorectal adenoma and 10 cancer samples (Fig. 4; Tables 1, 2). Firstly, we examined the expression levels of miR-124 and miR-133b in 20 paired samples. As shown in Tables 1 and 2, the expression levels of miR-124 and miR-133b in tumors (adenoma or cancer) were significantly down-regulated (ratio under 0.67) in 17 and in all cases, respectively, of these 20 patients. Interestingly, these miRs were already highly down-regulated in the adenomas. Next, we examined the protein expression levels of PKM isoforms in the cancer tumors samples by Western blot analysis. Expectedly, PKM2 expression was found to be dominant in all normal colon tissues compared to PKM1 (Fig. 4a and supplementary Fig S2a). Interestingly, the level of PKM2 expression was further elevated in all the tumor samples compared with that for the adjacent normal tissues (Fig. 4a and Supplementary Fig. S2a). Finally, we examined the expression of PKM1 and PKM2 in 7 clinical samples that were available for immunohistochemistry (IHC). In the crypt of normal colon tissues, a larger number of PKM2-immunocomplexes were found compared with those for PKM1 (Fig. 4b, c, and d). Also, in the tumor samples, more PKM2-immnocomplexes were found compared with those in the normal tissues neighboring the tumor in the same specimen (Fig. 4e, f, g, and h).

**Figure 5:**
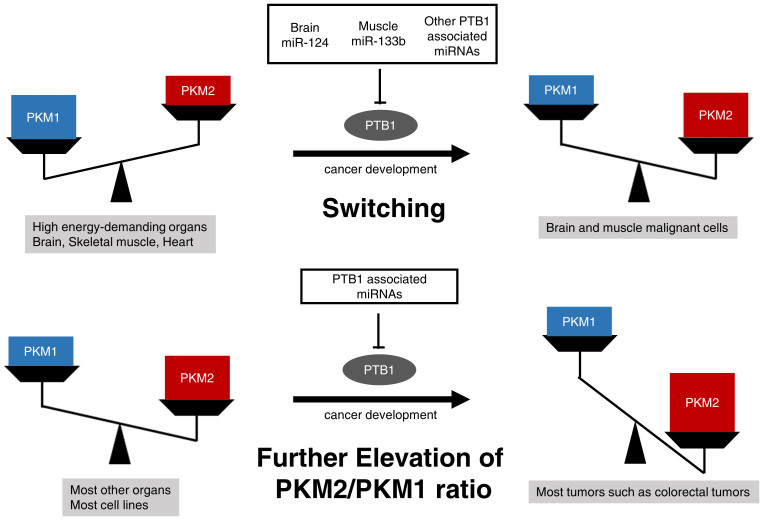
Schematic diagram of the function of *PTB1*-associated miRs (miR-124, miR-133b, and others) related to the expression of PKM1 and PKM2 in cancer development. Brain-specific microRNA (miR-124) and muscle-specific microRNA (miR-133b) up-regulate PKM1 by silencing PTB1 in high energy-demanding organs such as brain, skeletal muscle, and heart. PKM1 is dominant in only normal skeletal muscle, brain, and heart. *PTB1*-associated miRs such as miR-124 and miR-133b are down-regulated and switch PKM expression from PKM1 to PKM2 during cancer development in only these malignant cells. In other organs such as colon, PKM2 is dominant even in the normal tissue. In such organs, the *PKM2/PKM1* ratio is further elevated through the down-regulation of *PTB1*-associated miRs during cancer development. Other *PTB1*-associated miRs such as miR-1(muscle), miR-9 (brain), and miR-137 (brain) probably have the same functions.

These findings suggest that the ratio of PKM2/PKM1 in tumor samples was further elevated through the down-regulation of *PTB1*-associated miRNAs such as miR-124 and 133b during cancer development even in PKM2-dominant normal colon tissues. These findings taken together suggest that the alteration of PKM isoform expression during cancer development resulted in the switching from PKM1 to PKM2 (elevating the PKM2/PKM1 ratio) effected by *PTB1-*associated miRNAs (Fig. 5).

## Discussion

Much evidence indicates that PKM1 is expressed in normal differentiated tissues, whereas PKM2 is expressed in proliferating and cancer cells^18^,^21^. However, there are few data systematically demonstrating the expression of PKM isoforms in normal tissues. By using mass spectrometry, Bluemlein *et al.* reported that PKM2 instead of PKM1 is preferentially expressed in many normal differentiated tissues^22^. Also, using TCGA RNA-Seq datasets, Desai et al. reported that *PKM2* is found dominantly in normal tissues except for muscle and brain^23^. In our systematical study using 19 types of normal tissues from different organs, only brain, skeletal muscle, and heart expressed mainly *PKM1* rather than *PKM2*. These findings were also confirmed by the protein expression profile found for mouse tissue samples. Our results were thus similar to those reported by Bluemlein *et al* and Desai *et al*. These PKM1-dominant organs such as brain and muscle demand high energy to perform their functions; i.e., they express mainly PKM1 for use in the TCA cycle and thereby gain energy efficiently. In addition, these PKM1-dominant organs have machinery for the switch from PKM1 to PKM2 during cancer development. These are rather special cases. On the other hand, the organs in which the tissues have a short cell cycle, such as the gastrointestinal tract^37^, expressed mainly PKM2 rather than PKM1 even in normal tissues. IHC of normal colorectal tissue showed that PKM1 expression was dominant in the muscle layer, vascular smooth muscle, and Auerbach-Plexus. However, PKM2 dominated in the epithelial cells, which are the origin for cancer development (data not shown), which reflects the results of PKM2 expression in tumor samples from the patients. In the current study, we demonstrated that these PKM2-dominant organs underwent further elevation of their *PKM2/PKM1* ratio during cancer development by biochemical and histochemical analyses (Fig. 4).

Although it has already been proved that PKM switching during cancer development occurs in only special cases, the mechanism regulating PKM expression remained largely unknown. Also, at the single-cell level, the switching of PKM isoforms has not been observed yet. Desai et al. reported that PKM2 expression correlates with the hypomethylation status of intron1 of the PKM gene^23^, but this correlation is not isoform specific at the transcription level. Previously, it was reported that hnRNP proteins including PTB1 regulate PKM expression^21^. PTB1 repressively binds to the region flanking exon 9 in the PKM gene, resulting in exon 10 inclusion in the mRNA and up-regulation of PKM2 expression in cancer cells^6^,^7^. Previously, we found that miR-124 induced autophagy in colon cancer cells (data not shown). Therefore, we considered that miR-124 might regulate molecules correlated with energy metabolism, and finally we found that miR-124 mainly targeted PTB1. The tissue distributions of miR-124 were preferentially high in the brain, skeletal muscle, and heart, but fairly low in the gastrointestinal tract such as the stomach and colon. Therefore, we hypothesized that miRNAs such as miR-124 contributed to the fine tuning of PKM1 and PKM2 expression by regulating the PKM splicer PTB1. Furthermore, we found that miRNAs that target PTB1 consisted of muscle-specific miRNAs, such as miR-1 and miR-133 or brain-specific miRNAs such as miR-9, miR-124, and miR-137. It has been demonstrated that miRNAs regulate physiological responses, and so this machinery that regulates PKM expression by *PTB1*-associated miRNAs may be considered to be biologically significant in carcinogenesis. In addition, it is very reasonable that these *PTB1*-associated miRNAs would be down-regulated and that this down-regulation would be correlated with the switching of PKM1 to PKM2, further elevating the PKM2/PKM1 ratio during tumor development.

Our results strongly suggest that *PTB1*-associated miRNAs and PTB1 could be potential target molecules for the development of novel anti-cancer drugs or therapeutic or diagnostic markers in the near future.

## Methods

### Patients and samples

The study was reviewed and approved by the institutional review board of Gifu University Hospital (Gifu, Gifu, Japan) or Fujita Health University Hospital (Toyoake, Aichi, Japan) in accordance with the Declaration of Helsinki. All patients were informed of the investigational nature of the study and provided written informed consent before study enrollment. All human samples were obtained from patients who had undergone biopsy or surgery for resection between 2002 and 2014. Ten paired adenoma samples were obtained from Fujita Health University Hospital. Also, 10 paired cancer samples were obtained from Gifu University Hospital. All patients with previously untreated (or recently diagnosed) colorectal cancer were selected. The characteristics of the patients are shown in Tables 1 and 2. Under a pathologist's supervision, all tissue samples pairs were collected from surgically resected tissues, with these paired samples being from the primary tumor and its adjacent non-tumor mucosal tissue in the same patient.

### Mouse samples

Animal experimental protocols were approved by the Committee for Animal Research and Welfare of Gifu University. BALB/cSLC-nu/nu (nude) mice were obtained from Japan SLC (Hamamatsu, Japan). After a mouse had been killed, protein and RNA were extracted and used for Western blotting and real-time reverse transcription-PCR analysis, respectively. Details of the methods are given below in the Western blotting and Real-time reverse transcription-PCR sections.

### Cell culture

All cell lines were obtained from JCRB (Japanese Collection of Research Bioresources) Cell Bank. The medium used to culture human colon cancer cell line DLD-1 and neuroblastoma cell line NB-9 was RPMI-1640; that for human neuroblastoma cell line IMR-32, Dulbecco's modified Eagle's medium; that for human rhabdomyosarcoma cell line RD, Eagle's minimal essential medium; that for human rhabdomyosarcoma cell line KYM-1, a 1:1 mixture of Dulbecco's modified Eagle's medium and Ham's F12 medium. Other cell lines indicated in Figure 1a were cultured according to the cell bank's protocols. All media were supplemented with 10% (v/v) heat-inactivated FBS (Sigma-Aldrich Co, St. Louis, MO USA) and 2 mM L-glutamine under an atmosphere of 95% air and 5% CO_2_ at 37°C.

### Transfection experiments

All cells were seeded in 6-well plates at a concentration of 0.5 × 10^5^ per well (10–30% confluence) on the day before the transfection. The mature types of miR-124 and miR-133b (mirVana^TM^ miRNA mimic; Ambion, Foster City, CA, USA) or siRNAs for *PTB1* (Invitrogen Carlsbad, CA) were used for the transfection of the cells, which was achieved by using cationic liposomes, Lipofectamine^TM^ RNAiMAX (Invitrogen), according to the manufacturer's Lipofection protocol. The nonspecific control miRNA (HSS, Hokkaido, Japan) sequence was 5′-GUAGGAGUAGUGAAAGGCC-3′, which was used as a control for nonspecific effects^38^. The sequence of the mature type of miR-124 used in this study was 5′-UAAGGCACGCGGUGAAUGCC-3′; that of miR-133b, 5′-UUUGGUCCCCUUCAACCAGCUA-3′; and that of siR-*PTB1* for the 3′UTR region, 5′-AUCUCUGGUCUGCUAAGGUCACUUC-3′. The effects manifested by the introduction of miRs and siRNAs into the cells were assessed at 48 or 72 h after the transfection.

### Western blotting

Whole cells were homogenized in chilled lysis buffer comprising 10 mM Tris-HCl (pH 7.4), 1% NP-40, 0.1% deoxycholic acid, 0.1% SDS, 150 mM NaCl, 1 mM EDTA, and 1% Protease Inhibitor Cocktail (Sigma-Aldrich Co.) and stood for 20 min on ice. After centrifugation at 13,000 rpm for 20 min at 4°C, the supernatants were collected as whole-cell protein samples. Protein contents were measured with a DC Protein assay kit (Biorad, Hercules, CA, USA). Ten micrograms of lysate protein was separated by SDS-PAGE using 10.0 or 12.5% polyacrylamide gels, and electroblotted onto a PVDF membrane (PerkinElmer Life Sciences, Inc., Boston, MA, USA). After blockage of nonspecific binding sites for 1 h with 5% nonfat milk in PBS containing 0.1% Tween 20 (TBS-T), the membrane was incubated overnight at 4°C with primary antibodies. The next day, the membrane was then washed 3 times with TBS-T, incubated further with secondary antibodies at room temperature for 1 hour, and then washed 3 times with TBS-T. The immunoblots were visualized by use of Ammersham ECL Plus Western Blotting Detection Reagents (GE Healthcare, Buckinghamshire, UK). Primary antibodies used were as follow: anti-PTB1, (Cell Signaling Technology, Inc., Danvers, MA, USA); anti-PKM1 or PKM2 (Novus Biologicals, USA), and anti-β-actin antibody (Sigma-Aldrich Co). HRP-conjugated goat anti-rabbit and horse anti-mouse IgG (Cell Signaling Technology) were used as secondary antibodies. PKM1 and PKM2 expressions in various cells, normal tissues from mouse, and clinical samples were detected under the same experimental conditions at the same time. β-actin or GAPDH was used as an internal control.

### Real-time reverse transcription-PCR

Total RNA was isolated from cultured cells or tumor tissues by using a NucleoSpin microRNA isolation kit (TaKaRa, Otsu, Japan). RNA concentrations and purity were assessed by UV spectrophotometry. RNA integrity was checked by formaldehyde gel electrophoresis. To determine the expression levels of miR-124 and miR-133b, we conducted quantitative RT-PCR (qRT-PCR) by using TaqMan MicroRNA Assays (Applied Biosystems) and THUNDERBIRD Probe qPCR Mix (TOYOBO Co.,LTD.,Osaka Japan) according to the manufacturer's protocol. *RNU6B* was used as an internal control. For determination of the expression levels of *PTB1*, *PKM1*, *PKM2*, and glyceraldehyde-3-phosphate dehydrogenase (*GAPDH*) mRNAs, total RNA was reverse-transcribed with a PrimeScript® RT reagent Kit (TaKaRa). Real-time PCR was then performed with primers specific for them by using THUNDERBIRD SYBR qPCR Mix (TOYOBO). The primers for *PTB1, PKM1, PKM2, and GAPDH* were the following: *PTB1*-sense, 5′-ATC AGG CCT TCA TCG AGA TGC ACA-3′, and *PTB1*-antisense, 5′-TGT CTT GAG CTC CTT GTG GTT GGA-3′; *PKM1*-sense, 5′-CGA GCC TCA AGT CAC TCC AC-3′, and *PKM1*-antisense, 5′-GTG AGC AGA CCT GCC AGA CT-3′^24^; *PKM2*-sense, 5′-ATT ATT TGA GGA ACT CCG CCG CCT-3′, and *PKM2*-antisense, 5′-ATT CCG GGT CAC AGC AAT GAT GG-3′^24^; *GAPDH*-sense, 5′-CTC AGA CGG CAG GTC AGG TCC ACC-3′, and *GAPDH*-antisense, 5′-CCA CCC ATG GCA AAT TCC ATG GCA-3′. *GAPDH* was used as an internal control. The primers for both PKM1 and PKM2 were used after confirmation that efficient CT values had been obtained from standard curves. The relative expression levels were calculated by the ΔΔCt method.

### Luciferase reporter assay

Searching the Target Scan 6.2 database (http://www.targetscan.org/) to find algorithm-based binding sites of miR-124 or miR-133b, we found the predicted binding sites to be at position 329-336; miR-124 and 1031-1038; miR-133b in the 3′UTR of *PTB1* mRNA. The sequence region, containing the putative binding sequence of miR-124 and miR-133b, were inserted into a pMIR-REPORT^TM^ Luciferase miRNA Expression Reporter Vector (Applied Biosystems) according the manufacturer's protocol. Moreover, we made another pMIR construct encompassing a mutated seed sequence for miR-124 (Wild type; GT**GCC**TTA, mutant; GT**AAA**TTA) and miR-133b (Wild type; GG**ACC**AAA, mutant; GG**CAA**AAA) by using a PrimeSTAR® Mutagenesis Basal Kit (TaKaRa). The mutation of the vector was confirmed by sequence analysis. pRL-TK *Renilla* Luciferase Reporter vector (Promega, Madison WI, USA) was used as an internal control vector. DLD-1 were seeded into 96-well plates at a concentration of 0.1 × 10^4^ per well on the day before the transfection. DLD-1 cells were co-transfected with either reporter vector (0.01 μg/well each) and 20 nM miR-124, miR-133b or nonspecific non-coding siRNA (Dharmacon, Tokyo, Japan), which co-transfection was achieved by using Lipofectamine^TM^ RNAiMAX. Luciferase activities were measured at 24 h after co-transfection by using a Dual-Glo Luciferase Assay System (Promega) according to the manufacturer's protocol. Luciferase activities were reported as the firefly luciferase/*Renilla* luciferase ratio.

### Immunofluorescence study

We transfected DLD-1 cells with nonspecific control miRNA, miR-124 or miR-133b. At 48 h after the transfection, the cells were fixed for 15 min with 4% paraformaldehyde.They were then transferred to PBS and kept in it for 15 min, followed by exposure to 1% Block Ace (Dainippon Sumitomo Pharma Co., Ltd., Tokyo, Japan) for 20 min to block nonspecific antibody binding. Next, the cells were incubated with primary antibody against PKM1 or PKM2 (Novus Biologicals, USA) for 2 h at room temperature (RT). After a rinse in PBS for 15 min, the cells were then incubated with avidin-anti-rabbit antibody (Dako, Carpenteria, Calif, USA) for 60 min, followed by streptavidin-conjugated Alexa Fluor 555 (Molecular Probes, Eugene, OR, USA) for 30 min at RT. Finally, the samples were counterstained with DAPI for 15 min and observed under a fluorescence microscope.

### Histopathology and immunohistochemistry

For histopathology, clinical samples were stained with hematoxylin and eosin (H&E). The avidin-biotin complex method or EnVision+System-HRP (Dako, CA, USA) method was used for immunohistochemistry according to manufacturer's protocol. PKM1 and PKM2 (Novus Biologicals) were labeled on normal and tumor sections. We compared the staining intensity of PKM1 and PKM2 in normal sections. Also, we compared the staining intensity of PKM2 between normal and tumor areas in the same section in the same patient.

### Statistics

Each examination was performed in triplicate. In experiments on clinical samples, we calculated the relative ratio of each miRNA in tumor/adjacent normal tissues. We defined the expression levels > 1.5 were designated as up-regulation and those < 0.67 as down-regulation, which fold changes were obtained from the results of linear discriminant analysis of the miRNA expression patterns from many of our previous reports^39^,^40^,^41^. Statistical significances of differences were evaluated by performing the two-sided Student's *t*-test. The values were presented as the mean ± standard deviation. A *P* value < 0.05 was considered to be statistically significant.

## Author Contributions

K.T and Y.A. designed the research and wrote the paper. K.T., N.S., Y.I., Y.N., and Y.O. performed the experiments. K.T., N.S., M.K., H.S., N.Y., Y.N., Y.I., Y.O., K.U., and Y.A. analyzed the data. Y.N., T.S., Y.I., F.M., K.Y., Y.O., K.U., and Y.A. provided technical or material supports. Y.A. supervised the entire project.

## Supplementary Material

Supplementary Information Supplementary Information

## Figures and Tables

**Table 1:** Clinicopathological features and expression of miR-124 and miR-133b in colorectal adenoma

Case	Age	Sex^a^	Site^b^	Size^c^	Type^d^	Grade^e^	miR-124 T/N^f^	miR-133b T/N^g^
1	70	M	T	15	Ip	High	0.01	0.02
2	64	M	D	15	Ip	High	0.09	0.17
3	59	M	S	11	Ip	Low	0.46	0.16
4	44	F	S	10	Ip	Low	0.38	0.19
5	63	M	R	10	Isp	Low	0.25	0.52
6	31	M	A	10	Isp	Low	0.19	0.06
7	64	M	D	6	Isp	Low	0.43	0.32
8	44	F	S	19	Ip	Low	0.57	0.08
9	70	M	A	20	IIa	Low	0.78	0.12
10	59	M	S	10	Ip	Low	0.46	0.11

^a^M, male; F, female.

^b^Location of tumor; A, ascending colon; T, transverse colon; D, descending colon; S, sigmoid colon; R, rectum.

^c^Diameter in mm.

^d^Ip, pedunculate; Isp, subpedunculate; IIa, superficial-elevated.

^e^Low, low-grade adenoma (mild and moderate atypia); High, high-grade adenoma (severe atypia and carcinoma *in situ*).

^f^miR-124 relative ratio (tumor tissue/normal adjacent mucosa).

^g^miR-133b relative ratio (tumor tissue/normal adjacent mucosa).

**Table 2:** Clinicopathological features and expression of miR-124 and miR-133b in colorectal cancer

Case	Age	Sex^a^	Site^b^	Size^c^	Depth^d^	Stage^e^	miR-124 T/N^f^	miR-133b T/N^g^
1	72	F	A	35x32	SS	B	0.15	0.17
2	42	M	R	30x20	SS	B	0.19	0.63
3	53	F	R	45x40	SS	B	0.06	0.02
4	68	M	R	65x60	SE	D	1.76	0.01
5	59	F	S	60x50	SE	D	0.45	0.29
6	56	F	S	27x24	SS	C	0.36	0.14
7	82	F	A	33x28	MP	A	0.25	0.01
8	78	F	C	50x80	SI	C	0.28	0.02
9	52	M	S	20x25	MP	A	1.54	0.05
10	38	F	R	50x95	MP	A	0.03	0.07

^a^M, male; F, female.

^b^Location of tumor; C, cecum; A, ascending colon; S, sigmoid colon; R, rectum.

^c^Diameter in mm.

^d^MP, Mucosa propria; SS, Subserosa; SE, Serosa exposure; SI, Serosa invasion.

^e^Dukes' system.

^f^miR-124 relative ratio (tumor tissue/normal adjacent mucosa).

^g^miR-133b relative ratio (tumor tissue/normal adjacent mucosa).
