# Supplementary material for: Organ-specific PTB1-associated microRNAs determine expression of pyruvate kinase isoforms
Source: Sci Rep. 2015 Feb 27;5:8647. doi: 10.1038/srep08647 (PMC4342556; doi:10.1038/srep08647)
Supplement: Supplementary Information [file srep08647-s1.pdf]

Organ-specific *PTB1*-associated microRNAs determine expression of pyruvate kinase isoforms

Kohei Taniguchi,[1,2] Yuko Ito,[3] Nobuhiko Sugito,[1] Minami Kumazaki,[1] Haruka Shinohara,[1] Nami Yamada,[1] Yoshihito Nakagawa,[4] Tarou Sugiyama,[5] Manabu Futamura,[5] Yoshinori Otsuki,[3] Kazuhiro Yoshida,[5] Kazuhisa Uchiyama,[2] and Yukihiro Akao[1*]

[1] United Graduate School of Drug Discovery and Medical Information Sciences, Gifu University, 1-1 Yanagido, Gifu 501-1193, Japan

[2] Department of General and Gastroenterological Surgery, Osaka Medical College, Daigaku-cho, Takatsuki, Osaka 569-8686, Japan

[3]Department of Anatomy and Cell Biology, Division of Life Sciences, Osaka Medical College, 2-7 Daigaku-machi, Takatsuki, Osaka 569-8686, Japan

[4]Department of Gastroenterology, Fujita Health University, School of Medicine, Kutsukake-cho, Toyoake, Aichi 470-1192, Japan

[5]Department of Oncological surgery, Gifu University School of medicine, 1-1 Yanagido, Gifu 501-1193, Japan

Running title: Organ-specific *PTB1*-associated microRNAs and Warburg effect

Corresponding author: Kohei Taniguchi, M.D., United Graduate School of Drug Discovery and Medical Information Sciences, Gifu University, 1-1 Yanagido, Gifu 501-1193, Japan, Tel.: +81-58-230-7607, Fax: +81-58-230-7604, E-mail: sur144@poh.osaka-med.ac.jp

Supplementary Information

Supplementary Figure S1

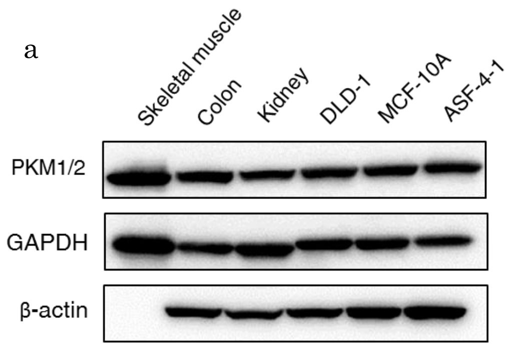

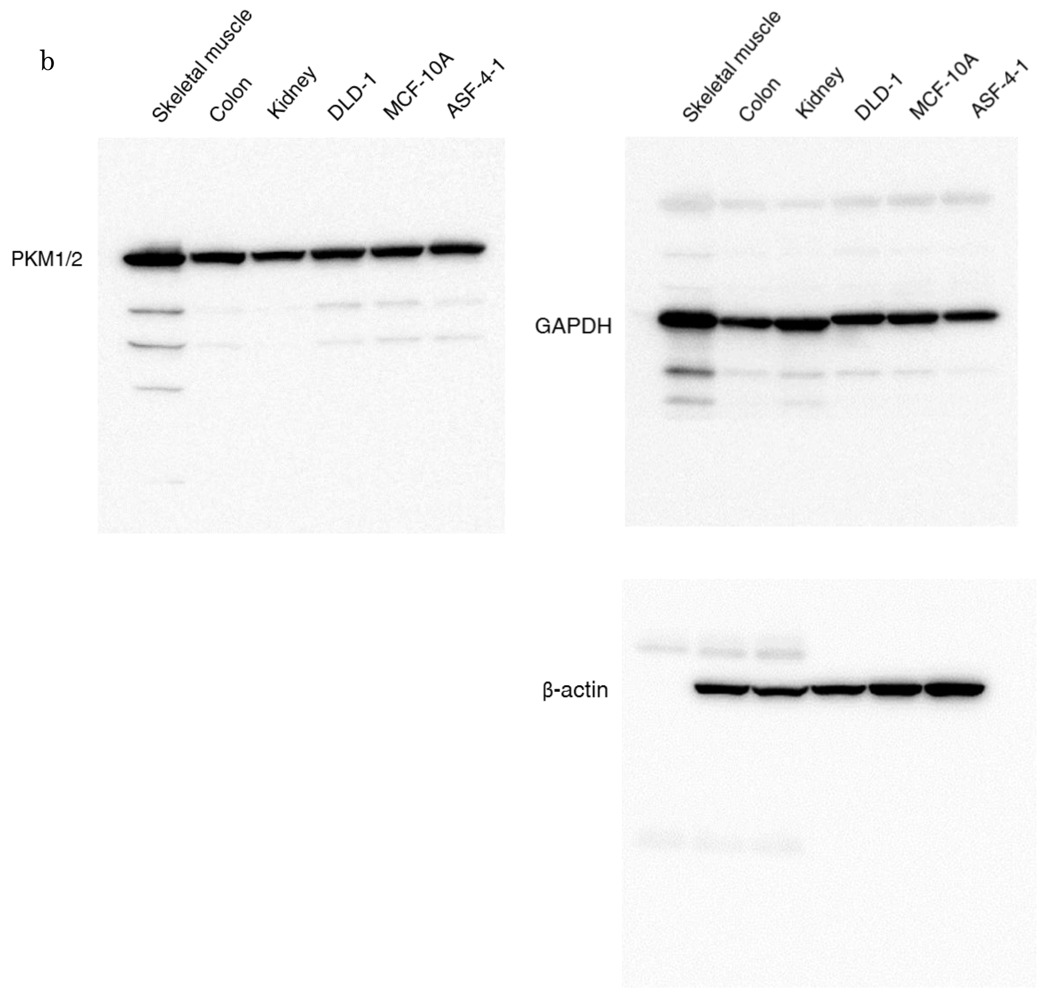

Supplementary Figure S2

a

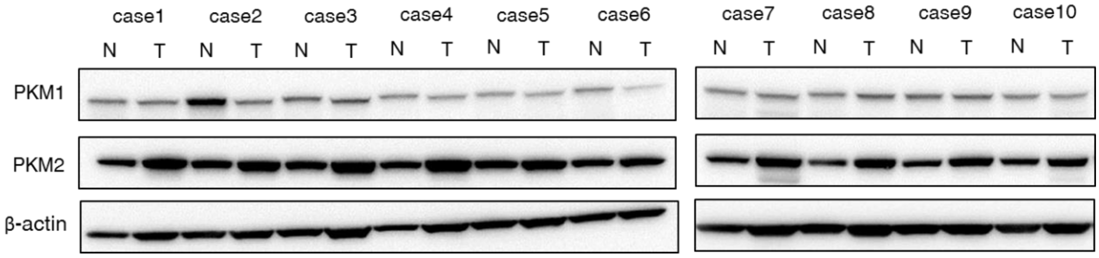

b

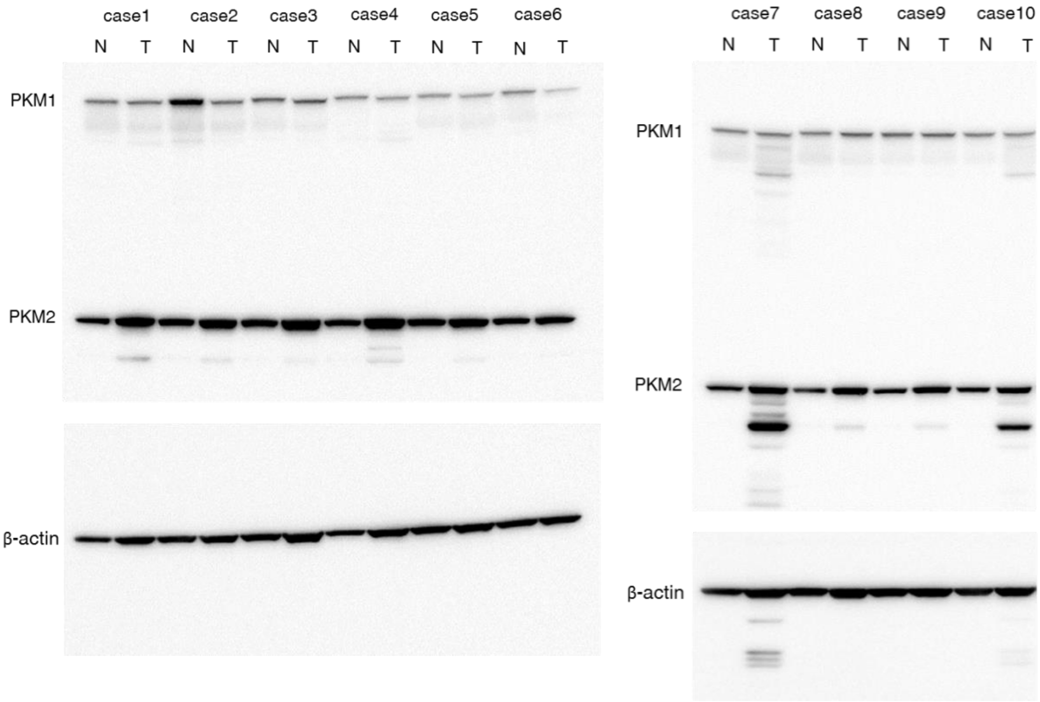

Supplementary Figure S3

a

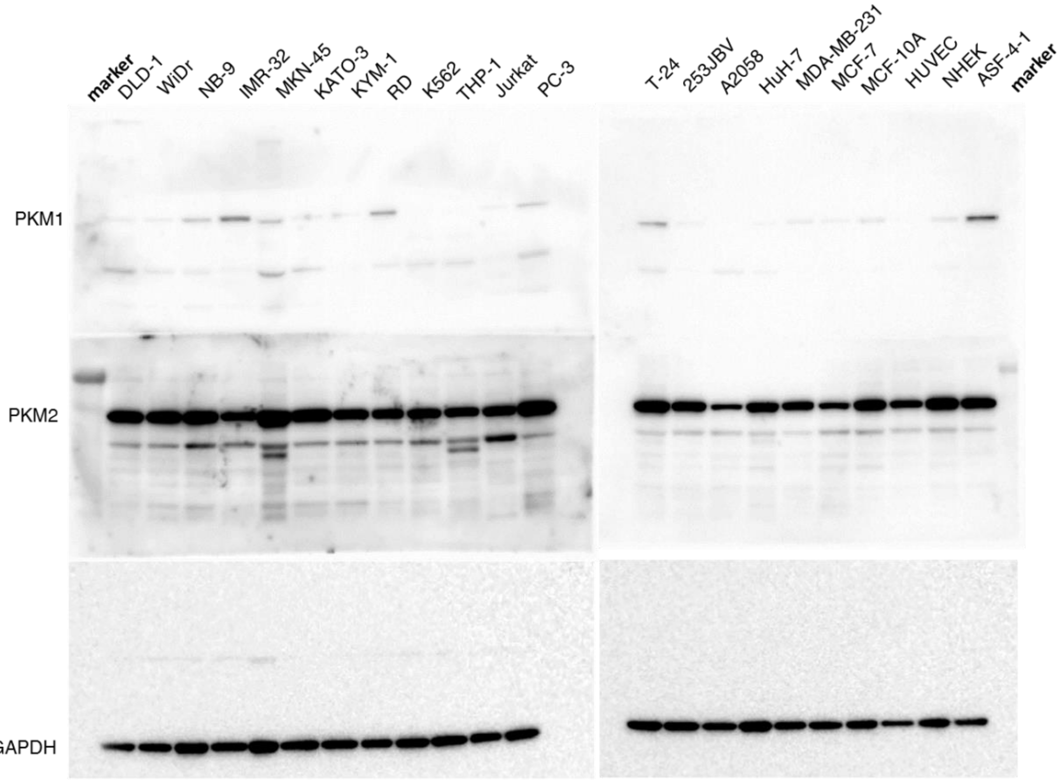

b

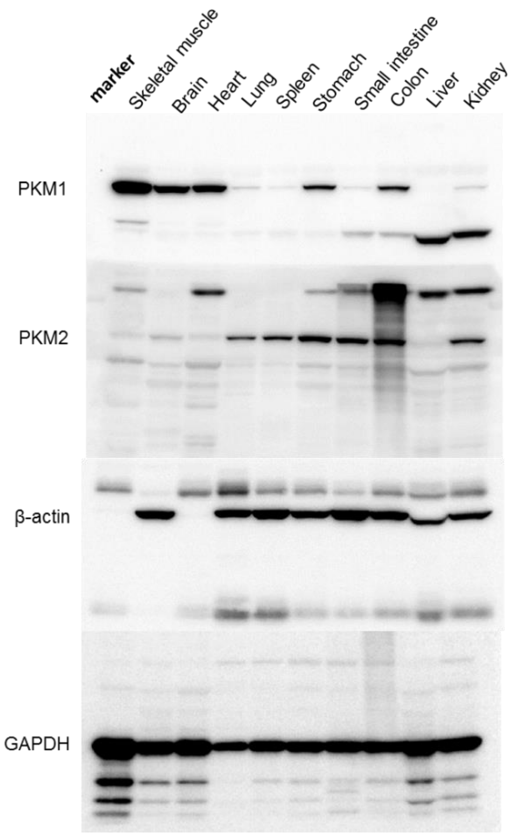

Supplementary Figure S4

a

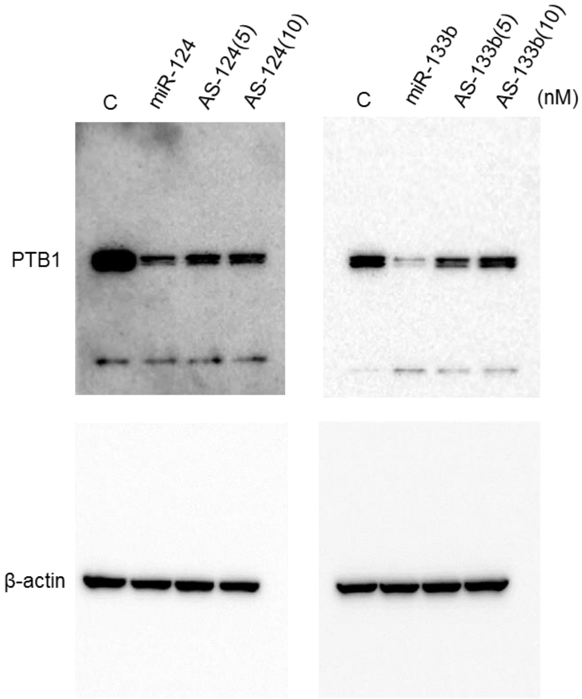

b

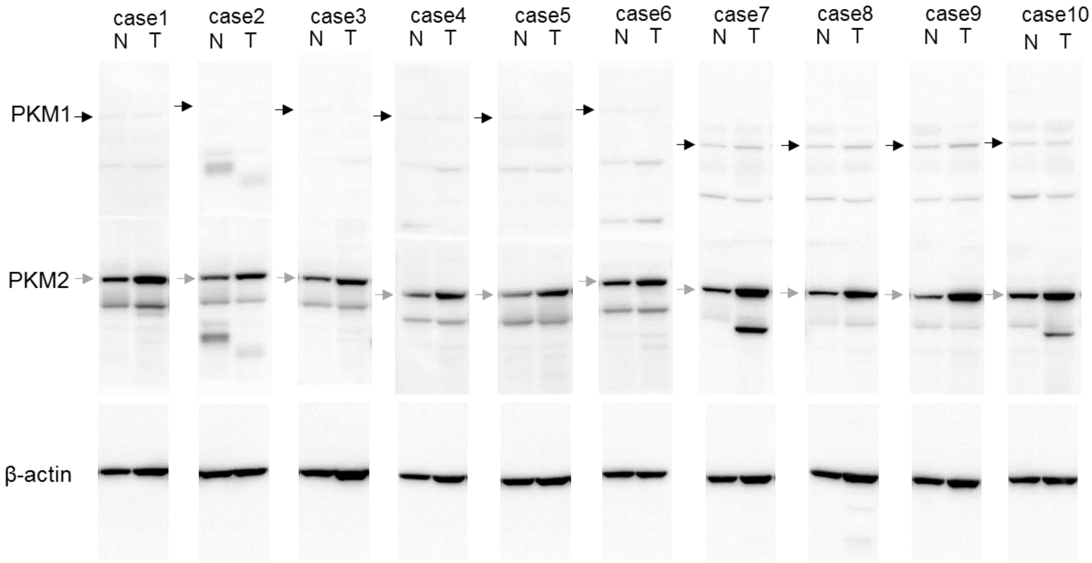

Supplementary Figure legends

Supplementary Figure S1. (a) The protein expression of PKM1/2 in some representative samples in Fig 1a and d of the manuscript. We selected skeletal muscle tissue, which seemed to be PKM1 dominant; colon, which seemed to be even in its expression levels of PKM1 and PKM2; and kidney, which seemed to be PKM2 dominant as representative mouse organ samples (as used in Fig. 1d). Also, we selected DLD-1 cells as representative cancer cells, MCF10A as a representative normal cell line, and ASF-4-1 cells which showed that the level of PKM1 was relatively high as representative primary cells in Fig. 1a of the manuscript. The primary antibodies used for PKM1/2 was purchased from Cell Signaling Technology, Inc., Danvers, MA, USA. (b) Full blots of "a."

Supplementary Figure S2. (a) The protein expression of PKM1 and PKM2 in clinical specimens of cancer tumor (T) and the adjacent normal tissues (N). PKM1 and PKM2 were detected by Western blotting in under the same experimental conditions at the same time. The primary antibodies used for PKM1 and PKM2 were purchased from Cell Signaling Technology, Inc., Danvers, MA, USA. (b) Full blots of "a."

Supplementary Figure S3. (a) Full blots of Figure 1a. (b) Full blots of Figure 1d.

Supplementary Figure S4. (a) Full blots of Figure 3f. (b) Full blots of Figure 4a.
